# Ultra‐Confined Phonon Polaritons and Strongly Coupled Microcavity Exciton Polaritons in Monolayer MoSi_2_N_4_ and WSi_2_N_4_

**DOI:** 10.1002/advs.202307691

**Published:** 2024-03-07

**Authors:** Juan Zhang, Yujie Xia, Lei Peng, Yiming Zhang, Ben Li, Le Shu, Yan Cen, Jun Zhuang, Heyuan Zhu, Peng Zhan, Hao Zhang

**Affiliations:** ^1^ The State Key Laboratory of Photovoltaic Science and Technology and School of Information Science and Technology and Department of Optical Science and Engineering and Key Laboratory of Micro and Nano Photonic Structures (MOE) Fudan University Shanghai 200433 China; ^2^ Department of Physics Fudan University Shanghai 200433 China; ^3^ National Laboratory of Solid State Microstructures Collaborative Innovation Center of Advanced Microstructures and School of Physics Nanjing University Nanjing 210093 China; ^4^ Yiwu Research Institute of Fudan University Chengbei Road Yiwu City Zhejiang 322000 China

**Keywords:** excitons, polaritons, polariton quantum condensate, 2D materials

## Abstract

The 2D semiconductors are an ideal platform for exploration of bosonic fluids composed of coupled photons and collective excitations of atoms or excitons, primarily due to large excitonic binding energies and strong light‐matter interaction. Based on first‐principles calculations, it is demonstrated that the phonon polaritons formed by two infrared‐active phonon modes in monolayer MoSi_2_N_4_ and WSi_2_N_4_ possess ultra‐high confinement factors of around ≈10^5^ and 10^3^, surpassing those of conventional polaritonic thin‐film materials by two orders of magnitude. It is observed that the first bright exciton possesses a substantial binding energies of 750 and 740 meV in these two monolayers, with the radiative recombination lifetimes as long as 25 and 188 ns, and the Rabi splitting of the formed cavity‐exciton polaritons reaching 373 and 321 meV, respectively. The effective masses of the cavity exciton polaritons are approximately 10^−5^ *m*_*e*_, providing the potential for high‐temperature quantum condensation. The ultra‐confined and ultra‐low‐loss phonon polaritons, as well as strongly‐coupled cavity exciton polaritons with ultra‐small polaritonic effective masses in these two monolayers, offering the flexible control of light at the nanoscale, probably leading to practical applications in nanophotonics, meta‐optics, and quantum materials.

## Graphical abstract

The phonon polaritons formed by two infrared‐active phonon modes in monolayer MoSi_2_N_4_ and WSi_2_N_4_ possess ultra‐high confinement factors of around 10^5^ and 10^3^, and the Rabi splittings of the formed cavity‐exciton polaritons reach 373 and 321 meV, respectively.

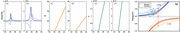

## 1 Introduction

The increase of electric field strength through trapping light at the nanoscale in optical microcavities results in strong light‐matter interactions, which leads to strong nonlinearity, large photonic forces, enhanced emission, and absorption probabilities, and importantly, the formation of new states of matter arising from the hybridization of elementary excitation of solid with photons, including surface plasmon, phonon and exciton polaritons, also known as different kinds of collective oscillations of polarization charges in matter. These polaritons encompass a wide range of electromagnetic spectrum from microwave to ultraviolet wavelengths.^[^ ^1^ ^]^ In recent years, polaritons have played an important role in developing next‐generation photonic and polaritonic applications, such as on‐chip light sources,^[^ ^2^ ^]^ quantum computing,^[^ ^3^ ^]^ optical transistors,^[^ ^4^ ^]^ Bose‐Einstein condensation (BEC),^[^ ^5^ ^]^ superfluidity,^[^ ^6^ ^]^ long‐range energy propagation,^[^ ^7^ ^]^ etc.

Recently, the phonon polaritons supported in van der Waals and 2D materials raise great interest, since they can be confined to ultra‐small volumes over a million times smaller than that of diffraction‐limited photons in vacuum. Generally two figures‐of‐merit are used to characterize the propagating phonon polaritons in real space: confinement factor and lifetime. Recent experiments reported measuring confinement factors/lifetimes of 60/9 ps in α −MoO_3_ flakes and 500/1.6 ps in hBN flakes, respectively.^[^ ^8^ ^]^ These confinement factors are comparable to those of graphene plasmon polaritons, but the lifetimes are nearly one order of magnitude larger than that of graphene plasmon polaritons. However, unlike the traditional phonon polaritons in 3D polar materials that arise from the splitting of longitudinal optical (LO) and transverse optical (TO) phonon modes, when the dimensionality crosses over to two dimensions, the screened macroscopic Coulomb interaction for 2D systems becomes inversely proportional to in‐plane wavevectors. This results in the absence of the LO‐TO splitting at the Γ point.^[^ ^9^ ^]^ Therefore, strictly speaking, the so‐called phonon polaritons cannot be well‐defined in polar monolayer materials.^[^ ^10^ ^]^ Arising from the fact that 2D LO phonon modes in monolayer materials are associated with ultra‐low‐loss and highly confined electromagnetic modes, the 2D LO phonon can be thus regarded as the 2D phonon polaritons, with properties similar to their 3D counterparts.^[^ ^11^ ^]^

By Coulomb interactions, excitons are bound states with the pairing of electrons and holes, which play an important role in determining the optical properties in semiconducting materials. Owing to the large exciton binding energy, the excitonic properties of 2D semiconductors have been widely studied, especially the family of transition metal dichalcogenides (TMDs).^[^ ^12^, ^13^, ^14^ ^]^ Furthermore, benefiting from the tunability of semiconductor microcavity,^[^ ^15^ ^]^ the coupling strength and other physical properties of microcavity exciton polaritons can be significantly improved. For some 2D systems placed in microcavities with strong light‐matter couplings, the Rabi splitting of exciton‐polaritons can be enhanced to 200 – 300 meV.^[^ ^16^, ^17^ ^]^

Here, we demonstrate the phonon and exciton polaritons of novel materials family monolayer MoSi_2_N_4_ and WSi_2_N_4_. We first examine the structural stability, electronic, transport, phonon vibrations, and optical properties. Our results show that the quasi‐particle (QP) bandgaps are renormalized to 3.20 and 3.03 eV considering spin‐orbit coupling (SOC) effect for monolayer MoSi_2_N_4_ and WSi_2_N_4_. We conduct an analysis of electron eigenvalues at the Γ point, considering factors such as atomic energy levels, crystal‐field splitting, and the impact of SOC effects. We observe a notably large confinement factor of phonon polaritons formed by two infrared‐active phonon modes in both monolayer MoSi_2_N_4_ and WSi_2_N_4_. We find that the excitons in two monolayers possess considerable large binding energies of 0.75 and 0.74 eV, respectively. By utilizing many‐body perturbation theory (MBPT), we explore exciton dynamics mechanisms, including electron–electron scatterings, electron–phonon scatterings, and intrinsic direct recombination. Theoretical calculations show that the Rabi splitting of monolayer MoSi_2_N_4_ and WSi_2_N_4_ reaches 373 and 321 meV for the first bright exciton, respectively.

## 2 Results and Discussion

### 2.1 Crystal Structures and Bandstructures

**Figure 1:**
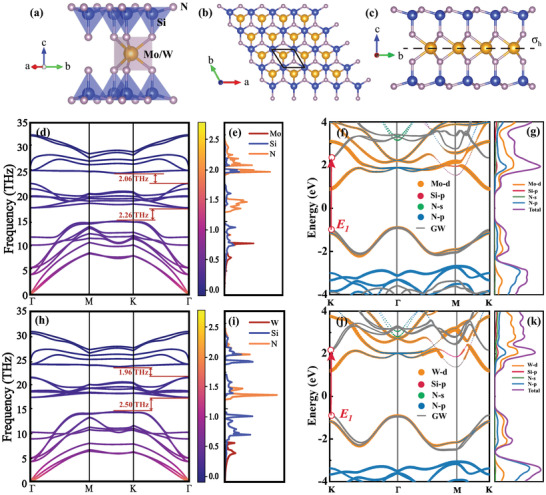
a) Crystal structure of MoSi_2_N_4_ and WSi_2_N_4_ monolayers. b) Top and c) side views. The phonon dispersions and phonon density of states of d,e) MoSi_2_N_4_ and h,i) WSi_2_N_4_. The project band structures and PDOS of f,g) MoSi_2_N_4_ and j,k) WSi_2_N_4_. The gray lines are *G*_0_ *W*_0_ band structure.

The crystal structures of monolayer MoSi_2_N_4_ and WSi_2_N_4_ are shown in **Figure** **1**a–c, which possess seven sub‐layers, belonging to the space group of P‐6m2 (No. 187) (point group *D*_3*h*_). The optimized lattice constants of monolayer MoSi_2_N_4_ are a = b = 2.89$\overset{˚}{A}$, while those of WSi_2_N_4_ are a = b = 2.90$\overset{˚}{A}$, as listed in **Table** **1**, which are in good agreement with the previous work.^[^ ^18^ ^]^ These two structures both possess an in‐plane mirror symmetry.

**Table 1:** Optimized lattice constants *a*, eigenvalues of excitons *E*_*x*_, high‐frequency dielectric constant ϵ_∞_ and Born effective charges Z*.

Material	*a*	*E*_*x*_ [eV]	$Z_{a}^{*}$ [e]	$Z_{b}^{*}$ [e]	$Z_{c1}^{*}$ [e]	$Z_{c2}^{*}$ [e]	ϵ_∞_	
MoSi_2_N_4_	2.89	2.45	0.89	3.31	−2.87	−0.87	5.46	
WSi_2_N_4_	2.90	2.29	1.40	−1.17	−2.84	3.31	4.64	

The calculated phonon dispersions with average phonon occupancy at 300 K described by the Bose‐Einstein statistics, with the consideration of the 2D implementation of the nonanalytical‐term correction^[^ ^9^ ^]^ for monolayer MoSi_2_N_4_ and WSi_2_N_4_ are shown in Figure 1d,h, which reveals that both phonon dispersions possess no imaginary frequencies, indicating that these two monolayers are thermally stable at low temperatures, and the optical phonon modes belonging to the twelve optical branches with largest frequencies are not well excited at room temperature in both monolayers. As shown in Figure 1d,h, two phonon bandgaps are observed up to 2.26 and 2.05 THz for monolayer MoSi_2_N_4_, and 2.50 and 1.96 THz for monolayer WSi_2_N_4_. By enforcing the rotational sum rules to the second force constants implemented in the Hiphive package,^[^ ^19^ ^]^ the calculated dispersion for ZA modes around the Γ point manifests quadratic shape, and those for TA and LA modes are linear.

Here to eliminate the spurious polarization field from the neighboring monolayer, which may influence the atomic movements within the monolayer, the Coulomb cutoff for the macroscopic dielectric screening is used here. Generally the relationship between LO and TO phonon frequencies influenced by the Coulomb screening effects can be written as,^[^ ^9^, ^10^ ^]^

(1) $$\omega_{\mathrm{LO}}^{2}-\omega_{\mathrm{TO}}^{2}=\frac{e^{2}{\left|q\right|}^{2}}{\Omega }W\left(q\right){\left|\hat{q}\cdot \sum_{n}Z_{n}^{*}\eta_{n}\right|}^{2}$$

where Ω is the unit‐cell volume, $\hat{q}$ is the unit vector of phonon momentum **q**, and $Z_{n}^{*}$ is the Born effective charge tensor of each atom in the unit‐cell. $\eta_{n}={\hat{e}}_{\beta ,n}/\sqrt{M_{n}}$ is the atom displacement along **q** direction, and ${\hat{e}}_{\beta ,n}$ is the phonon eigenvector, *M*_*n*_ is the mass of *n* ^*th*^ atom. *W*(*q*) is the screened macroscopic Coulomb interaction in the long wavelength limit. For 3D systems, since *W*(*q*)∝|*q*|^−2^, the LO‐TO splitting at Γ point exists only when the Born charge $Z_{n}^{*}$ is nonzero for polar materials. When the dimensionality is reduced to two dimensions, the 2D Coulomb‐screened interaction can be written as $W{\left(q\right)}^{2D}=\frac{1}{2q\varepsilon_{0}\varepsilon_{2D}}$, in which ϵ_2*D*_ is the in‐plane dielectric function of 2D material, which indicates that, there is no LO‐TO splitting at Γ point in 2D materials even for polar materials.

**Figure 2:**
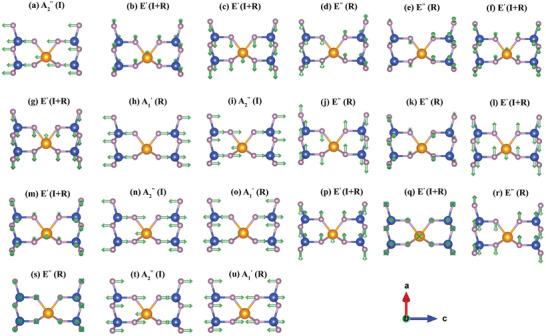
Vibration analysis for phonon modes at the Γ point of monolayer MoSi_2_N_4_.

To further investigate the phonon behaviors in these two monolayers, their vibrational modes are calculated and shown in **Figure** **2** and Figure S1 (Supporting Information), respectively. The small group at Γ point for monolayer MoSi_2_N_4_ and WSi_2_N_4_ are *D*_3*h*,_ which possesses six irreducible representation (irreps), and the vibrational modes at the Γ point can be decomposed by,

(2)

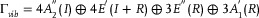




where *R*/*I* denotes Raman/Infrared activated modes. The vibrational modes for monolayer MoSi_2_N_4_ and WSi_2_N_4_ are similar due to their identical crystal symmetries.

The calculated electronic band structures based on the Perdew‐Bruke‐Ernzerhof (PBE) method projected by atomic orbitals for monolayer MoSi_2_N_4_ and WSi_2_N_4_ are shown in Figure 1f,j, which reveals that both monolayers possess indirect bandgaps with the values of 1.79 and 2.11 eV for MoSi_2_N_4_ and WSi_2_N_4_, respectively. The calculated partial density of states (PDOS) for these two monolayers are shown in Figure 1g,k, where Mo/W‐d and N‐p orbitals contribute dominantly around the conduction band minimum (CBM) located at *K* point, and Mo/W‐d orbital contributes dominantly around the valence band maximum (VBM) located at Γ point. When further considering the many‐body QP effects by using the one‐shot *G*_0_ *W*_0_+SOC method, the bandgaps increase to 3.20 and 3.03 eV, correspondingly. The QP energy can be obtained by expanding the electronic self‐energy Σ around the Kohn‐Sham eigenvalues with the first order,

(3) $$E_{nk}^{QP}=\varepsilon_{nk}+Z_{nk}\left[\Sigma_{nk}\left(\varepsilon_{nk}\right)-V_{nk}^{xc}\right]$$

where the normalization factor $Z_{nk}={\left[1-\frac{d\Sigma_{nk}\left(\omega \right)}{d\omega }{|}_{\omega =e_{nk}}\right]}^{-1}$, ε_*nk*_ is the single‐particle Kohn‐Sham (KS) energy of band index n. The self‐energy Σ_*nk*_ can be divided into the exchange item $\Sigma_{nk}^{x}$ and the energy‐dependent correlation item $\Sigma_{nk}^{c}\left(\omega \right)$, and $V_{nk}^{xc}$ is the exchange‐correlation potential for |*n* **k**〉. In Figure 1j, for monolayer WSi_2_N_4_, when the QP effects are considered, the position of VBM changes from Γ to *K* point, the value of $\Sigma_{nk}^{x}$ and $\Sigma_{nk}^{c}\left(\omega \right)$ are 1.38 and −17.04 eV at Γ points, 1.29 and −16.71 eV at *K* points, respectively. This is mainly due to the fact that K point possesses a larger total self‐energy correction term compared to Γ point on QP energy, which thus changes monolayer WSi_2_N_4_ into a semiconductor with a direct QP bandgap.

**Figure 3:**
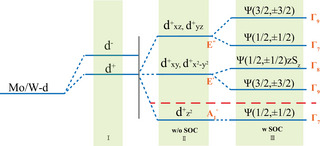
Schematic diagram of the evolution of the atomic Mo/W‐d and N‐p orbitals of monolayer MoSi_2_N_4_ and WSi_2_N_4_ into the valence and conduction bands, the red dotted line represents the Fermi energy level. Ψ(*j*, *m*_*j*_) means the basis functions that transform like an eigenstate of the angular momentum operator *J* of total angular momentum *J* and z component *m*_*j*_.

To better understand the contribution of atomic orbitals to electronic band structures for monolayer MoSi_2_N_4_ and WSi_2_N_4_, the crystal‐field splitting and SOC effects at Γ point are considered. According to project atomic orbital around CBM and VBM in Figure 1f,j, we take Mo/W‐d orbital into consideration, and the formation of the electronic energy around the Fermi level at Γ for these two monolayers can be summarized into three states, as shown in **Figure** **3**. At the stage (I), the chemical bonding separates the five‐fold degenerate Mo/W‐d atomic orbitals into the low‐energy |*d* ^+^〉 bonding states and high‐energy |*d* ^−^〉 antibonding states, and the +/− also means even/odd parity regarding the in‐plane mirror symmetric plane. At the stage (II), without the consideration of SOC effect (w/o SOC), the crystal‐field effect restricted by the *D*_3*h*_ point group, splits the fivefold degenerate low‐energy *d* ^+^ orbital into twofold degenerate ($d_{xz}^{+}$, $d_{yz}^{+}$) transforming with *E*″, and twofold degenerate ($d_{xy}^{+},d_{x^{2}-y^{2}}^{+}$) transforming with *E*′ for conduction band, and singly degenerate $d_{z^{2}}^{+}$ energy levels transforming with $A_{1}^{\prime }$ for valence band at Γ point. At stage (III), when the SOC effect is considered (w SOC), the irreps for the spinor is 2D Γ_7_ for double‐group *D*_3*h*_. As shown in Figure 3, the singly degenerate $|d_{z^{2}}^{+}\rangle$ is pushed down to |1/2,±1/2⟩ state transforming with Γ_7_ due to 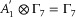. The twofold degenerate $|d_{xy}^{+},d_{x^{2}-y^{2}}^{+}\rangle$ is split into the |3/2,±3/2⟩ state transforming with Γ_9_ and the $\left|1/2,\pm 1/2\right\rangle zS_{z}$ state transforming with Γ_8_, with both Γ_8_ and Γ_9_ being 2D irreps, due to 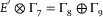. Similarly, the twofold degenerate $|d_{xz}^{+},d_{yz}^{+}\rangle$ is split into the |3/2,±3/2⟩ state transforming with Γ_9_ and the $\left|1/2,\pm 1/2\right\rangle zS_{z}$ state transforming with Γ_7_, due to 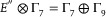.

### 2.2 Excitonic Behaviors and Excitonic Dynamics

In 2D materials, due to the enhanced quantum confinement effect and thus the reduction of Coulomb screening effects, the contributions from bound electron‐hole pairs called excitons generally play a key role in optical properties. The eigenstate for the *S* ^*th*^ electron‐hole pair can be obtained by solving the Bethe‐Salpeter equation (BSE),^[^ ^20^ ^]^

(4) $$\left(E_{ck}^{\mathrm{QP}}-E_{vk}^{\mathrm{QP}}\right)A_{vck}^{s}+\sum_{v^{\prime }c^{\prime }k^{\prime }}\langle vck\left|K^{\mathrm{eh}}\right|v^{\prime }c^{\prime }k^{\prime }\rangle A_{v^{\prime }c^{\prime }k^{\prime }}^{s}=\Omega_{S}A_{vck}^{S}$$

where $E_{vk,ck}^{\mathrm{QP}}$ denotes the QP eigenvalues for electronic |vk⟩ and |ck⟩ states, $A_{vck}^{s}$ is the envelop function for the *S* ^*th*^ exciton, the eigen state of which can be written as the summation over several electron‐hole pairs, i.e., $\left|S\right\rangle =\sum_{k,v,c}A_{vck}^{s}\left|ck\right\rangle ⊗\left|vk\right\rangle$, and |*v* **k**〉/|*c* **k**〉 is the constituent electron/hole pair with identical momenta. Ω^*S*^ is the exciton eigen energy. The contribution to the optical properties from the *S* ^*th*^ exciton is determined by its oscillator strength, which is defined as $f_{S}=\frac{{2|e\cdot \langle 0|v|S\rangle |}^{2}}{\Omega^{S}}$, where **e** is photon polarization vector, **v** is the velocity operator.

**Figure 4:**
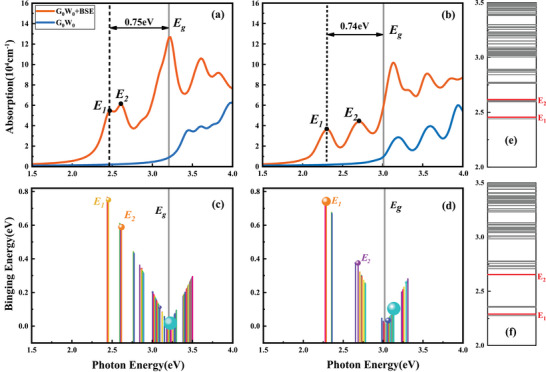
a,b) Energy‐dependent optical absorption of monolayers MoSi_2_N_4_ and WSi_2_N_4_ calculated by *G*_0_ *W*_0_+BSE (orange line) and *G*_0_ *W*_0_+RPA (blue line). c,d) Energy‐dependent excitons with different binding energies for monolayers MoSi_2_N_4_ and WSi_2_N_4_ with the circle radius representing oscillator strengths for respective excitons. e,f) Exciton energy levels for monolayers MoSi_2_N_4_ and WSi_2_N_4_.

**Figure 5:**
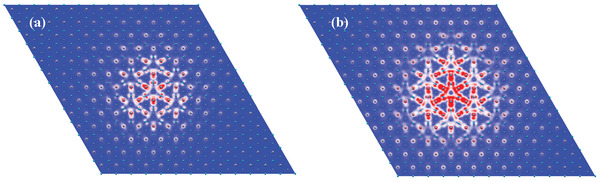
Exciton wavefuctions in real space of *E*_1_ exciton for monolayer a) MoSi_2_N_4_, b) WSi_2_N_4_, with the hole at center of supercells.

The calculated optical absorptions with and without consideration of the contribution from electron‐hole pairs denoted by orange lines and blue lines for monolayer MoSi_2_N_4_ and WSi_2_N_4_ are shown in **Figure** **4**a,b, respectively, which demonstrates that, in both monolayers, two excitonic absorption peaks can be observed in the QP‐bandgap region, labeled by *E*_1_ and *E*_2_, respectively. The eigenvalues of *E*_1_ and *E*_2_ are 2.45 and 2.61 eV for monolayer MoSi_2_N_4_, which are in good agreement with the experimental results of 2.21 eV, 2.35 eV^[^ ^21^ ^]^ and theoretical results.^[^ ^22^ ^]^ The excitonic absorption peaks for monolayer WSi_2_N_4_ are 2.29 eV and 2.66 eV, respectively. The calculated eigenvalues and the corresponding oscillator strength of excitons for these two monolayers are shown in Figure 4c,d, in which the oscillator strength is denoted by the radius of bubbles, which reveals that the two absorption peaks in Figure 4a,b are induced by the two bright excitons with large oscillator strengths, also denoted by *E*_1_ and *E*_2_, respectively. It is worth mentioning that the high oscillation intensity electron‐hole pairs (ionization continuum states) on the high‐energy side of the bandgap energy also contribute significantly to the absorptions in the high‐frequency region of monolayer MoSi_2_N_4_ and WSi_2_N_4_, but they are thermally unstable and will dissociate into free charge carriers at finite temperatures. The binding energies of the two bright excitons for these two monolayers, defined as the difference between the exciton eigen energy and the QP bandgap, are 0.75/0.59 and 0.74/0.37 eV, respectively, both being much larger than thermal fluctuation energy at room temperature of 26 meV, indicating that, *E*_1_ and *E*_2_ excitons for both monolayers are thermally stable at room temperature. In this work, we choose the first bright exciton *E*_1_ for the further study of the dynamics and light‐exciton interaction. The electron‐hole pairs with dominant contribution to *E*_1_ excitons for monolayer MoSi_2_N_4_ and WSi_2_N_4_ are shown in Figure 1f,g denoted by red arrows (at the K point), and the spatial spread for *E*_1_ excitons is shown in **Figure** **5**. Their estimated Bohr radii are 4.34 and 5.81 $\overset{˚}{A}$, respectively, indicating that *E*_1_ excitons in both monolayers are Wannier‐type excitons.

**Figure 6:**
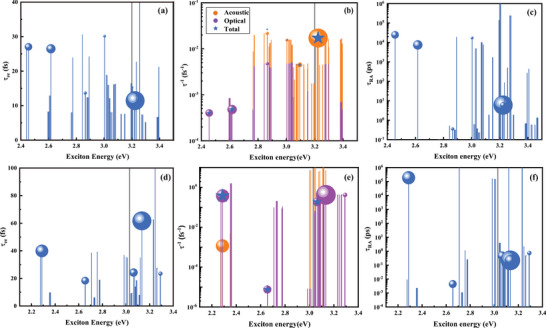
Exciton lifetime $\tau_{ee}^{S}$ induced by many‐body electron–electron interactions (a,d). Exciton‐phonon scattering rates at 77 K (b,e) and radiative lifetime (c,f) for monolayer MoSi_2_N_4_, WSi_2_N_4_.

In the following, we will discuss the exciton relaxation processes dominated by electron–electron (el–el), exciton‐phonon interactions (ex‐ph), and radiative recombination. The exciton decays induced by electron–electron interactions including quasi‐electrons and quasi‐holes produced by photoexcitations. Then the el–el lifetime for the *S* ^*th*^ exciton can be calculated according to $\tau_{ee}^{S}=(\sum_{vck}|A_{vck}^{S}{{|}^{2}\left(1/\tau_{ck}+1/\tau_{vk}\right))}^{-1}$, where the τ_*c* **k**_ and τ_*v* **k**_ represent the lifetimes of quasi‐electrons and quasi‐holes obtained from the self‐energy $\sum_{nk}\left(E_{nk}^{QP}\right)$, which is calculated based on the many‐body GW method. The results are shown in **Figure** **6**a,d, which indicates that most of the excitons within the QP bandgaps experience the el–el relaxation processes with a time scale ranging from about 5.0 to 40.0 fs, which are comparable to doped graphene of 40.0–80.0 fs.^[^ ^23^ ^]^

The interactions of excitons and lattice vibrations can be regarded as the superposition of electron‐ and hole‐phonon scattering processes. Based on the first‐order perturbation theory, the ex‐ph interactions Hamiltonian Δ*H* ^*ex* − *ph*^ can be written as,^[^ ^24^ ^]^

(5) $$\Delta H^{ex-ph}=\sum_{mn\nu ,Q,q}G_{mn\nu }\left(Q,q\right){\hat{c}}_{S_{m}\left(Q+q\right)}^{†}{\hat{c}}_{S_{n}\left(Q\right)}\left({\hat{d}}_{\nu q}+{\hat{d}}_{\nu -q}^{†}\right)$$

where $G_{mn\nu }$ is the ex‐ph coupling matrix element describing the transition probability from the initial *S*_*n*_(**Q**) exciton with momentum **Q** to final *S*_*m*_(**Q** + **q**) exciton with momentum **Q + q** of electron–phonon and hole‐phonon scattering processes, which can be obtained by Equation (23). ${\hat{c}}^{†}/{\hat{d}}^{†}$ and $\hat{c}/\hat{d}$ are the creation and annihilation operators for excitons/phonons, respectively. We take the two ex‐ph interaction processes of emission and absorption of phonons into consideration here. To simplify the model we consider that the exciton density *F*_*m* **Q + q**_ is far less than the phonon density *N*_ν**q**_, and only phonons with zero momentum are involved to calculate. The calculated ex‐ph scattering rates for both monolayers are shown in Figure 6b,e, which reveals that optical phonons scattering contributes the most compared to acoustic phonons to the *E*_1_ exciton in monolayer MoSi_2_N_4_ and WSi_2_N_4_. The lifetimes of ex‐ph coupling are 10 and 3 fs for *E*_1_ exciton in MoSi_2_N_4_ and WSi_2_N_4_, respectively, and the values are comparable with the 2D h‐BN around 15 fs.^[^ ^24^ ^]^

Finally, the calculated exciton radiative lifetimes $\tau_{RA}^{S}$ describing the direct recombination between electron and hole according to Equation (27), are shown in Figure 6c,f. The calculated $\tau_{RA}^{S}$ for *E*_1_ exciton of monolayer MoSi_2_N_4_ and WSi_2_N_4_ are 25 and 188 ns, respectively, which are much larger than those for black phosphorus of 200 ps,^[^ ^25^ ^]^ monolayer MoS_2_ of around 3 ns with doping.^[^ ^26^ ^]^ Considering the feasibility of experimental realization, the recombination lifetime is long enough for the experimental observations of exciton‐polaritons.

### 2.3 Phonon and Exciton Polaritons

Generally, the effective Hamiltonian for the interaction of Bose‐type quasiparticles (excitons and phonons herein) and photons can be expressed as^[^ ^27^ ^]^

(6) $$\begin{array}{ccc} H_{\text{interaction}} & = & \sum_{k}\hbar \nu_{k}a_{k}^{†}a_{k}+\sum_{k^{\prime }}E\left(k^{\prime }\right)b_{nk^{\prime }}^{†}b_{nk^{\prime }}\\ & & +\;i\hbar \sum_{k}g_{k}\left(b_{nk}^{†}a_{k}+b_{nk}a_{k}^{†}\right) \end{array}$$

where $a_{k}^{†}\left(a_{k}\right)$ and $b_{nk}^{†}\left(b_{nk}\right)$ are annihilation (creation) operators of Bose‐type quasiparticle phonon or excitons and photon fields with crystal wave vector of **k**, respectively. *g*_**k**_ is the coupling strength of quasiparticles and photons. Following the Bogoliubov‐transformation‐like method as follows^[^ ^27^ ^]^

(7) $$\begin{array}{c} a\left(k\right)=\sum_{\mu }\left[\alpha_{\mu }u_{\mu }\left(k\right)+\alpha_{\mu }^{†}\left(-k\right)v_{\mu }^{*}\left(-k\right)\right]\\ b_{n}\left(k\right)=\sum_{\mu }\left[\alpha_{\mu }u_{n\mu }\left(k\right)+\alpha_{\mu }^{†}\left(-k\right)v_{n\mu }^{*}\left(-k\right)\right] \end{array}$$

the interaction Hamiltonian can be diagonalized to the second‐quantization form as

(8) $$H=\sum_{\mu }E_{\mu }\alpha_{\mu }^{†}\left(k\right)\alpha_{\mu }\left(k\right)+\;\text{const.}\;$$

where *E*_μ_ is the eigenenergies of the hybrid quasiparticle called phonon or exciton polaritons, and $\alpha_{\mu }^{†}\left(\alpha_{\mu }\right)$ are annihilation (creation) operators of the hybrid polaritons.

#### 2.3.1 2D Phonon Polaritons

As is well known, in bulk polar materials, the long‐range polarization field induced by the optical‐phonon vibrations influences the phonon vibrations in turn, leading to the LO‐TO phonon splitting near the Γ point. On the other hand, the coupling between photons and Bose‐type phonons form the phonon polaritons as mentioned above, with the dispersion‐relation equation written as,^[^ ^28^ ^]^

(9) $$\frac{c^{2}}{\omega^{2}}k^{2}=\varepsilon_{\infty }\frac{\omega_{\mathrm{LO}}^{2}-\omega \left(\omega +i\gamma \right)}{\omega_{\mathrm{TO}}^{2}-\omega \left(\omega +i\gamma \right)}$$

where ω_*LO*/*TO*_ represents the LO/TO phonon frequency and γ is phonon damping rate. The Reststrahlen band is formed between LO and TO phonon frequencies, and there are two dispersion curves for such phonon polaritons, usually called as upper polariton band (UPB) and low polariton band (LPB). When the wave vector *k* is large enough, the LPB is dominated by the lattice vibration, and the UPB is dominated by photons. When *k* → 0, the situation is on the opposite.

When the dimensionality is reduced to two dimensions, the LO‐TO splitting at the Γ point giving rise to the formation of phonon polaritons is absent according to Equation (1) resulting from the 2D dynamical Coulomb screening effects, even for polar monolayers. Strictly, the phonon polaritons are not well defined and therefore absent in any 2D materials. Despite the absence of well‐defined phonon polaritons in 2D materials, recent reports demonstrated that,^[^ ^10^, ^11^ ^]^ the 2D LO phonon modes in the 2D polar materials manifest strongly confined evanescent electromagnetic modes, which is similar to the surface phonon polaritons in 3D systems. Therefore, in this way, the 2D LO phonon modes can be regarded as the equivalence of the surface phonon polaritons, also called 2D phonon polaritons, which was verified previously in 2D hBN^[^ ^11^ ^]^ and MoS_2_.^[^ ^9^ ^]^

In 2D materials, by considering the longitudinal phonon distribution to the ionic displacement and then using the conductivity within the random‐phase approximation (RPA) method, the lattice optical conductivity in the long‐wavelength limit (q → 0) can be written as,^[^ ^10^, ^11^ ^]^

(10) $$\sigma \left(\omega \right)=\frac{-i\omega /\Omega }{\omega_{TO}^{2}-\omega^{2}-2i\gamma \omega }{\left|\hat{q}\cdot \sum_{n}Z_{n}^{*}\eta_{n}\right|}^{2}$$

Here, we consider the monolayer to be sandwiched by a superstrate of permittivity ϵ_+_ and a substrate of permittivity ϵ_−_, and ϵ_*env*_ = (ϵ_+_ + ϵ_−_)/2. Then, the dispersion equation can be reduced to its quasistatic limit without consequential loss of accuracy, i.e., *q* = 2*i*ωϵ_0_ϵ_env_/σ(ω). Thus, the dispersion equation for 2D phonon polariton can be written as,^[^ ^10^, ^11^ ^]^

(11) $$q=\frac{-2\Omega \varepsilon_{0}\varepsilon_{\mathrm{env}}\left(\omega_{TO}^{2}-\omega^{2}-2i\gamma \omega \right)}{{\left|\hat{q}\cdot \sum_{n}Z_{n}^{*}\eta_{n}\right|}^{2}}$$

The calculated Born effective charges Z* for ions in both monolayers are listed in Table 1. The LO phonon group velocities *v*_*g*_ for monolayer MoSi_2_N_4_ and WSi_2_N_4_, defined from the microscopic parameters by $v_{g}={\left|\hat{q}\cdot \sum_{n}Z_{n}^{*}\eta_{n}\right|}^{2}/4\varepsilon_{0}\varepsilon_{env}\omega_{TO}\Omega$, are calculated and *v*_*g*_ for these two 2D materials are listed in **Table** **2**, in which *c* is the light speed in vacuum. As mentioned above, there exist two infrared‐active LO phonon modes in monolayer MoSi_2_N_4_ and WSi_2_N_4_ with the frequencies of 18.21, 25.72 THz, and 18.09, 24.97 THz, respectively, which possess nonzero effective charges, therefore they can contribute to the ionic part of optical conductivities.

**Table 2:** The LO phonon frequencies, the deceleration factor *D* and phonon damping rate γ for two infrared‐active LO/TO phonon modes in monolayer MoSi_2_N_4_ and WSi_2_N_4_, one infrared‐active LO/TO phonon modes in monolayer hBN.

MoSi_2_N_4_			WSi_2_N_4_			hBN^[^ ^11^ ^]^		
LO [THz]	D [c]	γ [cm^−1^]	LO [THz]	D [c]	γ [cm^−1^]	LO [THz]	D [c]	γ [cm^−1^]
18.21	6.3 × 10^−7^	10.7	18.09	4.9 × 10^−7^	8.3	41.3	1.7 × 10^−5^	1.17
25.72	1.4 × 10^−5^	18.3	24.97	1.0 × 10^−5^	16.6

The deceleration factor *D* (= *v*_*g*_/*c*) for these two infrared‐active 2D phonon polaritons in both monolayers are extremely low with 5−7 orders lower than light speed, also lower by 0−2 orders than those in monolayer h‐BN,^[^ ^11^ ^]^ and values of *D* for low‐frequency LO phonons in these two monolayers are comparable to that in monolayer MoS_2_.^[^ ^9^ ^]^ The phonon damping rates γ for all 2D phonon polaritons are calculated by only considering three‐phonon processes by using the Boltzmann transport theory implemented in the ShengBTE package,^[^ ^29^ ^]^ and similar calculations were previously reported in ref. [30]. The calculated γ values at room temperature for the two infrared‐active 2D phonon polaritons in monolayer MoSi_2_N_4_ and WSi_2_N_4_ are 10.7, 18.3 cm^−1^ and 8.3, 16.6 cm^−1^, respectively, as listed in Table 2, which are comparable to those in SrTiO_3_ and KTaO_3_, but larger than those in LiNbO_3_ and h‐BN,^[^ ^11^ ^]^ probably due to the heavier Mo and W atoms.

**Figure 7:**
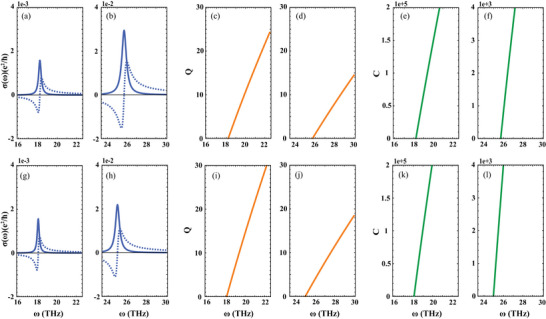
Properties of phonon polariton of monolayer MoSi_2_N_4_ and WSi_2_N_4_. a,b,g,h) Real (Re σ, solid lines) and imaginary (Im σ, dashed lines) parts of the conductivity σ of 2D MoSi_2_N_4_ and WSi_2_N_4_. c,d,i,j) Propagation‐quality factor, Q = Im σ /Re σ. e,f,k,l) Confinement factor, *C* = *q*/*q*_0_.

The electronic conductivities contributed by the infrared‐active LO phonon modes can be calculated according to Equation (10), and the calculated real (Re σ) and imaginary (Im σ) parts of the conductivity σ for monolayer MoSi_2_N_4_ and WSi_2_N_4_ are shown in **Figure** **7**a,b,g,h, respectively, with the mode damping rates listed in Table 2. The linewidth for the peak values of σ(ω) as shown in Figure 7a,b,g,h is proportional to γ, and the peak values of σ for the LO phonon modes with highest frequencies are larger than those with lower frequencies by one order.

The propagation‐quality factors *Q*, defined as *Q* = Im σ/Re σ, measuring the distance that the 2D phonon polariton travels before decaying in number of wavelengths, are calculated and shown in Figure 7c,d,i,j, respectively, which reveals that the *Q* shows slower decrease with increasing frequencies for 2D phonon polaritons with larger frequencies and larger damping rates γ, indicating that the quicker leakage of energy leads to smaller *Q*. Similar behavior can be observed for all 2D phonon polaritons irrespective of MoSi_2_N_4_ or WSi_2_N_4_. The *Q* tendency for monolayer MoSi_2_N_4_ and WSi_2_N_4_ are comparable to those in KTaO_3_, but slower than those in SrTiO_3_, LiNbO_3_, and h‐BN,^[^ ^11^ ^]^ probably due to their relatively larger damping rate γ.

Then the confinement factors *C* for 2D phonon polaritons, defined as *C* = *q*/*q*_0_ = λ_0_/λ_*ph*_ with λ_*ph*_ the wavelength for 2D phonon polariton, which is a measure for the compression of light wavelength λ trapped within a 2D phonon polariton with respect to light wavelength in free space λ_0_, are calculated and shown in Figure 7e,f,k,l, respectively. The calculated confinement *C* for all 2D phonon polaritons in these two monolayers is in the magnitude of 10^3^ and 10^5^, i.e. *C* ≈ *O*(10^3^) and *O*(10^5^), in which the higher value is larger than those in h‐BN by two orders, probably due to the smaller deceleration factors *D* for the lower frequency LO phonon in these two monolayers as listed in Table 2.

In addition, in order to consider the applied monolayer on top of the substrate, the influence of compressive/tensile strains are considered. The calculated band structures and phonon dispersions by applying the strains of −4, −2, +2, +4% along *a*‐axis of the two monolayers are shown in Figures S4 and S5 (Supporting Information). When the strains range from ‐2% to +2%, the PBE bandgaps are 2.02, 1.52 eV and 2.32, 1.89 eV for monolayer MoSi_2_N_4_ and WSi_2_N_4_, respectively, and the phonon frequency gradually redshifts. The calculated phonon properties include the deceleration factor *D* and phonon damping rate γ with the consideration of ±2% strains are shown in Table S1 (Supporting Information), and the optical conductivity σ, propagation‐quality factor Q and confinement factor C for monolayer MoSi_2_N_4_ and WSi_2_N_4_ are shown in Figure S6 (Supporting Information). Benefiting from the smaller phonon damping rate, the optical conductivity increases by an order of magnitude after applying strain, and the Q value also doubles. Besides, as shown in the Figure S8 and Table S1 (Supporting Information), the properties of the 1T‐phase monolayer MoSi_2_N_4_ and WSi_2_N_4_ are similar to the 2H‐phase ones under investigations here, but the 1T‐phase monolayers will not be discussed in details here.

#### 2.3.2 Exciton Polaritons

Due to the large binding energies of both *E*_1_ excitons in monolayer MoSi_2_N_4_ and WSi_2_N_4_ of 0.75 and 0.74 eV, as is shown in Figure 4a,b, which guarantee the thermal stabilities of *E*_1_ excitons against the thermal fluctuation at room temperature, and their long radiative recombination times of 25 and 188 ns, respectively as shown in Figure 6c,f, the *E*_1_ excitons in both monolayers are beneficial for the realization of exciton polaritons and polariton condensation when they are strongly coupled to the cavity photons. Generally, the total exciton energy *E*_*X*_(**k**) for excitons with finite momentum of **k** can be given as the summarization of energy of zero‐momentum transverse exciton (*E*_*X*0_) and the kinetic energy, i.e., *E*_*X*_(**k**) = *E*_*X*0_ + ℏ^2^ *k* ^2^/2*M*_*ex*_, with *M*_*ex*_ the mass of exciton given by *M*_*ex*_ = *m*_*e*_ + *m*_*h*_. The light dispersion in vacuum can be expressed as $\omega_{0}=ck/\sqrt{\varepsilon_{\infty }}$. For simplification, the influence of the kinetic energy term is neglected for low‐frequency cases under investigation. The transverse exciton energies *E*_*X*0_ for *E*_1_ excitons in 2D MoSi_2_N_4_ and WSi_2_N_4_ are 2.45 and 2.29 eV, respectively, as shown in Figure 4c,d. For enhancement of the exciton‐cavity photons coupling, assuming the monolayer MoSi_2_N_4_ and WSi_2_N_4_ are placed in the middle of the cavity with air (*n* = 1) filled in the space between the distributed Bragg reflectors (DBR), the dispersion for the exciton polaritons can be described from the coupled‐oscillator Hamiltonian model as follows,

(12) $$\left(\begin{array}{cc} E_{X}-E_{\mu } & g\\ g & \hbar \omega_{0}-E_{\mu } \end{array}\right)=0$$

where *g* is the coupling strength for exciton‐photon interaction, also called the Rabi coupling strength, and $\hbar g_{S}\approx \sqrt{\frac{2\pi E_{X}^{S}\mu_{S}^{2}}{n^{2}L\Omega }}$, with $g_{S}/E_{X}^{S}/\mu_{S}^{2}$ the Rabi coupling strength/eigen energy/the square modulus of the exciton transition dipole per unit cell for the *S* ^*th*^ exciton, *n* ^2^ = ϵ_*env*_, and Ω the area for 2D unit cell. The Rabi splitting for the *S* ^*th*^ exciton can be obtained as Δ*E* = 2*g*. The solution to Equation (12) is written as,

(13) $$E_{\mu }^{\pm }=\frac{E_{X}+\hbar \omega_{0}}{2}\mp \sqrt{g^{2}+\frac{{\left(E_{X}-\hbar \omega_{0}\right)}^{2}}{4}}$$

**Figure 8:**
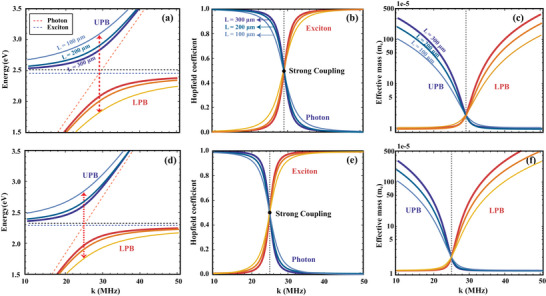
For cavity length of 100, 200, and 300 µm, (a,d) UPB and LPB branches for exciton polariton dispersions of monolayer MoSi_2_N_4_ and WSi_2_N_4_. b,e) Exciton and photon Hopfield coefficients for LPB branches in monolayer MoSi_2_N_4_ and WSi_2_N_4_. c,f) The effective masses of UPB and LPB for monolayer MoSi_2_N_4_ and WSi_2_N_4_.

**Figure 9:**
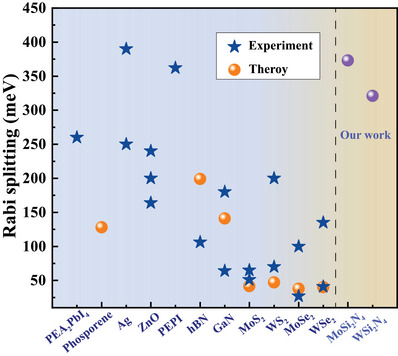
Comparisons of theoretical and experimental Rabi splitting values for monolayer MoSi_2_N_4_ and WSi_2_N_4_ with PEA_2_PbI_4_,^[^ ^16^ ^]^ Phosporene,^[^ ^31^ ^]^ Ag,^[^ ^32^, ^33^ ^]^ ZnO,^[^ ^34^, ^35^, ^36^ ^]^ PEPI,^[^ ^37^ ^]^ hBN,^[^ ^38^, ^39^ ^]^ GaN,^[^ ^38^, ^40^, ^41^ ^]^ MoS_2_,^[^ ^38^, ^42^, ^43^ ^]^ WS_2_,^[^ ^17^, ^38^, ^44^ ^]^ MoSe_2_,^[^ ^38^, ^45^, ^46^ ^]^ WSe_2_.^[^ ^38^, ^47^, ^48^ ^]^

Subsquently, based on Equation (12), the dispersion relation for exciton polaritons in monolayer MoSi_2_N_4_ and WSi_2_N_4_ with the length *L* of 100, 200, 300 µm with air filled in the space between the DBRs are calculated, and the results are shown in **Figure** **8**a,d, which demonstrate that both monolayers possess lower polariton band (LPB) and upper polariton band (UPB) as functions of wave vector **k** and quasiparticle energy, corresponding to ∓ in Equation (13), respectively. When the *L* is 300 µm, the calculated Δ*E* for monolayer MoSi_2_N_4_ and WSi_2_N_4_ are 373 and 321 meV, respectively. For comparison, we also calculate their dispersions for exciton polaritons by considering the exciton dipole field,^[^ ^49^ ^]^ i.e., $\frac{\hbar^{2}c^{2}k^{2}}{E_{\mu }^{2}}=\varepsilon_{\infty }+\frac{\mu_{S}^{2}/\Omega }{1-{\left(E_{\mu }/E_{X}^{S}\right)}^{2}}$, with ϵ_∞_ the high‐frequency constant, and the calculated Δ*E* are approximately equal to 334 and 319 meV at wave vectors of 29 and 25 MHz for these two monolayers. The comparison of theoretical and experimental Rabi splittings for different materials is shown in **Figure** **9**, which reveals that the Rabi splittings for monolayer MoSi_2_N_4_ and WSi_2_N_4_ are larger than those of PEA_2_PbI_4_,^[^ ^16^ ^]^ ZnO,^[^ ^34^, ^35^, ^36^ ^]^ GaN,^[^ ^38^, ^40^, ^41^ ^]^ hBN,^[^ ^38^, ^39^ ^]^ MoS_2_,^[^ ^38^, ^42^, ^43^ ^]^ WS_2_,^[^ ^17^, ^38^, ^44^ ^]^ MoSe_2_,^[^ ^38^, ^45^, ^46^ ^]^ WSe_2_,^[^ ^38^, ^47^, ^48^ ^]^ and Phosporene,^[^ ^31^ ^]^ but comparable to bulk (C_6_H_5_C_2_H_4_NH_3_)_2_PbI_4_ (PEPI)^[^ ^37^ ^]^ and Ag,^[^ ^32^, ^33^ ^]^ manifesting their superiority in terms of interaction between light and excitons. Furthermore, when the length of cavity decreases from 300 to 200 µ*m*, the Rabi splitting is changed to be 457 and 393 meV, and further decreases to 100 µ*m*, the Rabi splitting is increased to 646 and 556 meV, respectively, indicating stronger light‐matter interactions for smaller cavity lengths.

The calculated properties of exciton and exciton polariton with the consideration of ±2% strains are shown in Figure S7 (Supporting Information). The GW bandgap of −2, +2% strain for monolayer MoSi_2_N_4_ are 3.7 and 3.1 eV, and the values are 3.6 and 3.2 eV for WSi_2_N_4_, which both increase, compared with those without strains. The Rabi splitting are 327, 185 and 255, 226 meV for the two monolayers, respectively, which are smaller than those without strains.

To manifest the respective contribution from photons and excitons to the formation of exciton polaritons, the annihilation operator for exciton polariton, i.e., α_μ_ in Equation (8), can be written as a linear combination of annihilation operators for photon and exciton,^[^ ^50^ ^]^

(14) $$\begin{array}{cc} & \alpha_{LP\mu }\left(k\right)=\eta_{C}\left(k\right)b\left(k\right)+\eta_{X}\left(k\right)a\left(k\right)\\ & \alpha_{UP\mu }\left(k\right)=\eta_{C}\left(k\right)b\left(k\right)-\eta_{X}\left(k\right)a\left(k\right) \end{array}$$

where the prefactors η_*C*_(**k**) and η_*X*_(**k**) are known as Hopfield coefficients for cavity photons and excitons, respectively. The squares of the Hopfield coefficients in each UPB and LPB give the fraction of exciton and photon in exciton polariton, and the Bose commutation relation yields η_*C*_(**k**)^2^ + η_*X*_(**k**)^2^ = 1. When the strong coupling occurs between exciton and photon, the square of the Hopfield coefficient of the exciton and photon is equal to 0.5, which means that the exciton polariton is formed by half photon and half exciton, also corresponding to the location where the Rabi splitting occurs, i.e., at wave vectors of 29 and 25 MHz as shown in Figure 8b,e. With the increase of the wave vector, the Hopfield coefficient of the exciton η_*X*_(**k**) increases, but that for cavity photon η_*C*_(**k**) decreases, which means that, the LPB‐branch polaritons become more exciton‐like, whereas the UPB‐branch polaritons become more photon‐like. When the wave vector decreases, the situation is the opposite.

The polariton effective mass of LPB/UPB can be written as the weighted harmonic mean of the masses of its constituent exciton and photon as follows,^[^ ^51^ ^]^

(15) $$\begin{array}{cc} & \frac{1}{m_{LP}}=\frac{\eta_{C}^{2}}{m_{C}}+\frac{\eta_{X}^{2}}{M_{X}}\\ & \frac{1}{m_{UP}}=\frac{\eta_{C}^{2}}{M_{X}}+\frac{\eta_{X}^{2}}{m_{C}} \end{array}$$

where *m*_*C*_ is the effective mass for the DBR cavity photons with the value ≈10^−5^ *m*_*e*_, and *M*_*X*_ ≫ *m*_*C*_. The calculated polariton effective masses for polaritons in LPB/UPB branches are shown in Figure 8c,f, which show that, around the strong‐coupling location, i.e., $\eta_{C}^{2}=\eta_{X}^{2}=0.5$, both *m*_*LP*_ and *m*_*UP*_ are approximately equal to 2.04 × 10^−5^ *m*_*e*_ with the *L* of 300 µm, and they change in the range from 1 × 10^−5^ *m*_*e*_ to 5 × 10^−3^ *m*_*e*_ for different wave vectors, well smaller than the exciton effective mass *M*_*X*_ of 6.5 *m*_*e*_ for MoSi_2_N_4_ and 2.8 *m*_*e*_ for WSi_2_N_4_. The much smaller effective masses of UPB/LPB polaritons make possible the very high critical phase‐transition temperatures for macroscopic Bose‐Einstein condensation (BEC) of polaritons, denoted as *T*_*c*_. This is due to the relationship $T_{c}\propto \frac{n^{2/3}}{m_{UP/LP}}$, which theoretically exceeds those in counterpart systems formed by excitons by three to five orders, making it an ideal platform for achieving high‐temperature macroscopic quantum condensates.

Strictly speaking, the lifetime of polaritons τ_*LP*, *UP*_ is determined by the Rabi coupling strength *g*, lifetimes of cavity photons due to the imperfect mirrors τ_*C*_, and lifetimes of excitons τ_*X*_ mentioned above, and in microcavities with Γ_*X*_ ≪ Γ_*C*_ ≪ *g* where Γ_*X*, *C*_ ($=\frac{\hbar }{2\tau_{X,C}}$) is the decay rate of cavity photons and excitons, respectively, the lifetime of quantized exciton polariton can be expressed as a linear combination of cavity photon lifetime and exciton radiative lifetime,^[^ ^51^ ^]^ i.e., $1/\tau_{LP}=\eta_{X}^{2}/\tau_{X}+\eta_{C}^{2}/\tau_{C}$ and $1/\tau_{UP}=\eta_{C}^{2}/\tau_{X}+\eta_{X}^{2}/\tau_{C}$. As shown in Figure 6c,f, the calculated lifetimes of the first bright excitons τ_*X*_ for monolayer MoSi_2_N_4_ and WSi_2_N_4_ are 25 and 188 ns, respectively. Generally, the lifetimes of the cavity photon τ_*C*_ are 1‐10 ps. Therefore, $\tau_{LP}\approx \tau_{C}/\eta_{C}^{2}$ and $\tau_{LP}\approx \tau_{C}/\eta_{X}^{2}$ and at the location where the Rabi splitting occurs, τ_*UP*, *LP*_ ≈ 2τ_*C*_, with the time scale of ps.

## 3 Conclusion

In summary, based on the first principle and many‐body perturbation theory, we theoretically analyzed 2D phonon vibration mode and exciton dynamic processes of monolayer MoSi_2_N_4_ and WSi_2_N_4_. We have demonstrated the crystal‐field splitting and SOC effects at Γ point. Then we examine the properties of two infrared active phonon polaritons. The propagation‐quality factors *Q* tendency for monolayer MoSi_2_N_4_ and WSi_2_N_4_ are comparable to those in KTaO_3_ and the confinement factors C are larger than those in h‐BN by zero to two orders. Besides, we extensively demonstrate the 2D exciton polaritons of monolayer MoSi_2_N_4_ and WSi_2_N_4_. The large binding energy of the first bright exciton are 750 and 740 meV, and the calculated long exciton radiative lifetime is 25 and 188 ns, respectively, which is beneficial for the implementation of cavity exciton polaritons. We have calculated the strong Rabi splitting of the first bright exciton, the values are 373 and 321 meV, larger than many conventional polariton materials.

## 4 Experimental Section

The calculations were based on the first principles implemented using the Vienna Ab Initio simulation package (VASP) and PHONOPY. The 2D implementation of the nonanalytical‐term correction was performed to get the correct dispersion of the LO phonon by Quantum ESPRESSO. For the calculations on electronic structures and structural optimization, the Generalized gradient approximation (GGA) in the PBE parametrization was used to describe the exchange‐correlation potentials. A 21 × 21 × 1 Gamma‐centred reciprocal‐space grid was used for the K‐points sampling. A convergent 24 × 24 × 1 k‐mesh was used to calculate QP states for monolayer MoSi_2_N_4_ and WSi_2_N_4_, based on the many‐body interaction *G*_0_ *W*_0_ method implemented in the YAMBO software package. The kinetic cutoff energy of the plane waves was set to be 90 Ry.

Furthermore, many‐body perturbation theory was used to perform the electron correlation calculations. The Hamiltonian considering interacting electrons system can be expressed as the following Dyson equation,^[^ ^52^ ^]^

(16) $$\left[-\frac{1}{2}\nabla^{2}+V_{ion}+V_{H}+V_{xc}^{DFT}+\left(\Sigma {\left(E^{QP}\right)}_{nk}-V_{xc}^{DFT}\right)\right]\psi_{nk}^{QP}=E_{nk}^{QP}\psi_{nk}^{QP}$$

where V_*H*_ is the Hartree term, $V_{xc}^{DFT}$ is the DFT exchange‐correlation potential, $E_{nk}^{QP}$ and $\psi_{nk}^{QP}$ represent QP eigen‐energies and eigen‐wavefunctions, respectively. And the self‐energy operator Σ describes electron–electron interactions, which can be approximately obtained by Σ ≈ *iG*_0_ *W*_0_,^[^ ^52^, ^53^ ^]^

(17) $$\Sigma \left(r,r^{\prime };\varepsilon_{n}\right)=\frac{i}{2\pi }\int d\omega e^{i\omega 0^{+}}G_{0}\left(r,r^{\prime };\omega +\varepsilon_{n}\right)W_{0}\left(r,r^{\prime };\omega \right)$$

where the Green's function G_0_ is constructed by KS orbitals, and W_0_ is the frequency‐dependent dynamically screened interaction defined as *W*_0_ = [1 − *VP*_0_]^−1^ *V*, where P_0_ and V represent polarizability and bare Coulomb interaction, respectively.

The imaginary part of the electron self‐energy Σ_*n* **k**_ represents the lifetime due to the quasi‐particle effect, and the QP lifetime for |*n* **k** > electron is calculated according to

(18) $$\frac{1}{\tau_{nk}}=\frac{2}{\hbar }J\Sigma_{nk}\left(E_{nk}^{QP}\right)$$

Due to the weak Coulomb screening effect in 2D systems, the electron‐hole (exciton) interactions were enhanced significantly. The contribution from the electron‐hole pair of monolayer MoSi_2_N_4_ and WSi_2_N_4_ should be involved in the calculation of optical properties. We further solve the Bethe‐Salpeter equation (BSE) for the excitonic state,

(19) $$\left(E_{ck}^{QP}-E_{vk}^{QP}\right)A_{vck}^{S}+\sum_{v^{\prime }c^{\prime }k^{\prime }}\langle vck\left|K^{eh}\right|v^{\prime }c^{\prime }k^{\prime }\rangle A_{v^{\prime }c^{\prime }k^{\prime }}^{S}=\Omega_{S}A_{vck}^{S}$$

here *K* ^*eh*^ includes the kernel of electron‐hole interaction, Ω_*S*_ and $A_{vck}^{S}$ represent the eigen‐energies and momentum‐space envelop function for the *S*_*th*_ exciton. The real space wave function for the Sth exciton can be expressed as

(20) $$\Psi \left(r_{e},r_{h}\right)=\sum_{k,v,c}A_{vck}^{S}\psi_{k,c}\left(r_{e}\right)\psi_{k,v}^{*}\left(r_{h}\right)$$

Then, the imaginary part of the dielectric function ϵ_2_ can be calculated by summarizing over certain number of excitons,^[^ ^54^ ^]^

(21) $$\varepsilon_{2}\left(\omega \right)=\frac{16\pi^{2}e^{2}}{\omega^{2}}\sum_{S}{\left|e\cdot \langle 0|v|S\rangle \right|}^{2}\delta \left(\omega -\Omega_{S}\right)$$

where $e\cdot \left\langle 0|v|S\right\rangle =\Sigma_{vck}A_{vck}^{S}\left\langle vk|v|ck\right\rangle$, with **v** representing the velocity operator of the incident photons. In this work, six lowest QP conduction bands and six QP valence bands were used.

Similar to equation, the lifetime limited by the electron–electron interactions for the *S* ^*th*^ exciton can be written as,^[^ ^23^ ^]^

(22) $$\frac{1}{\tau_{ee}^{S}}=\sum_{vck}{\left|A_{vck}^{S}\right|}^{2}\left(\frac{1}{\tau_{ck}}+\frac{1}{\tau_{vk}}\right)$$

Furthermore, the relaxation process via phonons for excitons involves electron–phonon interactions, and the electron–phonon matrix element for the transition between the electron |*n* **k**〉 and |*m* **k** + **q**〉 via the phonon |**q** *v*〉 can be calculated by,

(23) $$g_{mnv}\left(k,q\right)=\langle \psi_{mk+q}\left|\partial_{qv}V\right|\psi_{nk}\rangle$$

And the exciton phonon matrix elements $G_{mnv}\left(Q,q\right)$ can be expressed as,^[^ ^24^ ^]^

(24) $$\begin{array}{c} G_{mnv}\left(Q,q\right)=\sum_{k}\left[\sum_{vcc^{\prime }}A_{vk,c^{\prime }\left(k+Q+q\right)}^{S_{m}\left(k+Q\right)*}A_{vk,c(k+Q)}^{S_{n}\left(Q\right)}g_{cc^{\prime }v}\left(k+Q,q\right)-\sum_{cvv^{\prime }}A_{v(k-q),c(k+Q)}^{S_{m}\left(k+Q\right)*}A_{v^{\prime }k,c\left(k+Q\right)}^{S_{n}\left(Q\right)}g_{vv^{\prime }v}\left(k-q,q\right)\right] \end{array}$$

where $A_{vk,c^{\prime }\left(k+Q+q\right)}^{m}$ and $A_{vk,c(k+Q)}^{n}$ are the exciton envelop functions. Using Matsubara's method, the imaginary part of exciton‐phonon self‐energy can be calculated,

(25) $$\begin{array}{ccc} J\Xi_{nQ} & = & -\frac{\pi }{N_{q}N_{k}}\sum_{m\nu q}{\left|G_{mnv}\left(Q,q\right)\right|}^{2}\left[\left(N_{vq}+1+F_{mQ+q}\right)\times \delta \left(E_{nQ}-E_{mQ+q}-\hbar \omega_{vq}\right)\right.\\ & & \left.+\left(N_{vq}-F_{mQ+q}\right)\times \delta \left(E_{nQ}-E_{mQ+q}+\hbar \omega_{vq}\right)\right] \end{array}$$

where N_vq_ and F_mQ+q_ are phonon and exciton occupations and N_q_ and N_k_ are the numbers of q points and k points. Using free exciton approximation, the self‐energy is reduced as,^[^ ^55^ ^]^

(26) $$\begin{array}{cc} J\Xi^{\text{Free}\;}= & -\frac{\pi }{N_{k}}\sum_{k}\sum_{\lambda }\sum_{vc}{\left|A_{vc}^{S}\right|}^{2}\{\\ & \;\sum_{c^{\prime \prime }}{\left|g_{cc^{\prime \prime }\lambda }\left(k\right)\right|}^{2}\left[N_{B}\left(\omega_{\lambda }\right)\delta \left(\varepsilon_{c}-\varepsilon_{c^{\prime \prime }}+\omega_{\lambda }\right)+\left(N_{B}\left(\omega_{\lambda }\right)+1\right)\delta \left(\varepsilon_{c}-\varepsilon_{c^{\prime \prime }}-\omega_{\lambda }\right)\right]\\ & +\left.\sum_{v^{\prime \prime }}{\left|g_{vv^{\prime \prime }\lambda }\left(k\right)\right|}^{2}\left[\left(N_{B}\left(\omega_{\lambda }\right)+1\right)\delta \left(\varepsilon_{v}-\varepsilon_{v^{\prime \prime }}+\omega_{\lambda }\right)+N_{B}\left(\omega_{\lambda }\right)\delta \left(\varepsilon_{v}-\varepsilon_{v^{\prime \prime }}-\omega_{\lambda }\right)\right]\right\} \end{array}$$

The excitons may experience the direct recombination decay which can be estimated as follows,^[^ ^56^ ^]^

(27) $$\gamma_{S}\left(Q\to 0\right)=\tau_{S}\left(Q\to 0\right)=\frac{2e^{2}E_{S}\mu_{S}^{2}}{\varepsilon_{0}S_{0}\hbar^{2}c}$$

where $\mu_{S}^{2}$ is the velocity dipole matrix element expressed by,

(28) $$\mu_{S}^{2}=\frac{\hbar^{2}}{m_{e}^{2}E_{S}^{2}}\frac{{\left|\langle G\left|p_{∥}\right|\Psi_{S}\left(0\right)\rangle \right|}^{2}}{N_{k}}$$

where *m*_*e*_ is the electron mass, 〈*G*|*p*_‖_|Ψ_*S*_(0)〉 is the momentum dipole matrix element in the exciton basis, and *N*_*k*_ is the number of **k** vector used in the calculation.

## Conflict of Interest

The authors declare no conflict of interest.

## Supporting information

Supporting Information

## Data Availability

The data that support the findings of this study are available from the corresponding author upon reasonable request.
