# Supplementary material for: Ultra‐Confined Phonon Polaritons and Strongly Coupled Microcavity Exciton Polaritons in Monolayer MoSi2N4 and WSi2N4
Source: Adv Sci (Weinh). 2024 Mar 7;11(18):2307691. doi: 10.1002/advs.202307691 (PMC11095159; doi:10.1002/advs.202307691)
Supplement: Supplementary file 1 — Supporting Information [file ADVS-11-2307691-s001.pdf]

ADVANCED SCIENCE
Open Access

Supporting Information

for *Adv. Sci.*, DOI 10.1002/advs.202307691

Ultra-Confined Phonon Polaritons and Strongly Coupled Microcavity Exciton Polaritons in Monolayer $MoSi_2N_4$ and WSi_2N_4

*Juan Zhang, Yujie Xia, Lei Peng, Yiming Zhang, Ben Li, Le Shu, Yan Cen, Jun Zhuang, Heyuan Zhu, Peng Zhan and Hao Zhang**

Supplementary Information

Ultra-confined Phonon Polaritons and Strongly Coupled Microcavity Exciton Polaritons in Monolayer $MoSi_2N_4$ and WSi_2N_4

Juan Zhang,[1] Yujie Xia,[1] Lei Peng,[1] Yiming Zhang,[1] Ben Li,[1] Le Shu,[1]
Yan Cen,[2] Jun Zhuang,[1] Heyuan Zhu,[1] Peng Zhan,[3] and Hao Zhang[1,4,*]

[1]*School of Information Science and Technology and Department of Optical Science and Engineering and Key Laboratory of Micro and Nano Photonic Structures (MOE), Fudan University, Shanghai 200433, China*

[2]*Department of Physics, Fudan University, Shanghai 200433, China*

[3]*National Laboratory of Solid State Microstructures, Collaborative Innovation Center of Advanced Microstructures and School of Physics, Nanjing University, Nanjing 210093, China*

[4]*Yiwu Research Institute of Fudan University, Chengbei Road, Yiwu City, Zhejiang 322000, China*

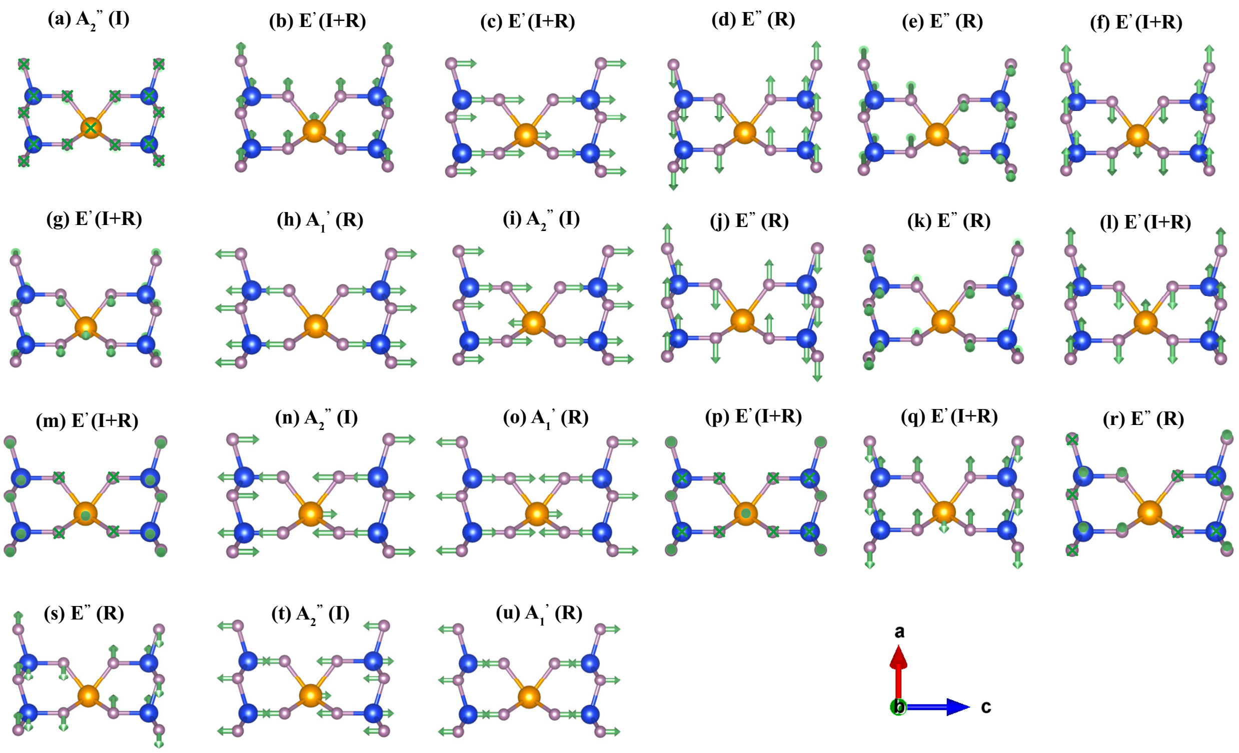

FIG. S1. Vibration analysis for phonon modes at the Γ point of monolayer WSi_2N_4.

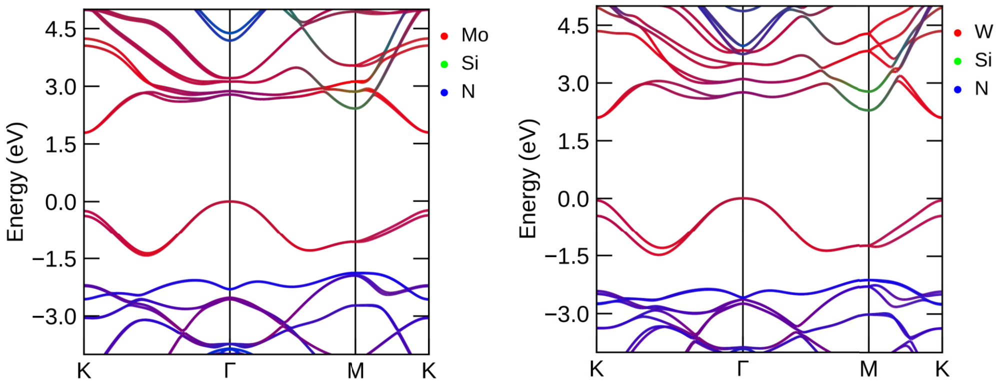

FIG. S2. The calculated band structures of monolayer WSi_2N_4 and WSi_2N_4 using PBE+SOC method.

* zhangh@fudan.edu.cn

index	mode	frequency(THz)		I/R
		$MoSi_2N_4$	WSi_2N_4	
1	ZA	0	0	I
2	TA	0	0	I+R
3	LA	0	0	I+R
4	TO	3.95	3.94	R
5	LO	3.95	3.94	R
6	TO	5.17	4.64	I+R
7	LO	5.17	4.64	I+R
8	ZO	8.39	8.62	R
9	ZO	11.29	10.22	I
10	TO	16.39	16.95	R
11	LO	16.39	16.95	R
12	TO	18.21	18.09	I+R
13	LO	18.21	18.09	I+R
14	ZO	20.44	20.47	I
15	ZO	20.64	20.74	R
16	TO	25.72	24.97	I+R
17	LO	25.72	24.97	I+R
18	TO	25.74	24.99	R
19	LO	25.74	24.99	R
20	ZO	31.49	30.67	I
21	ZO	32.01	31.51	R

FIG. S3. Vibration modes for phonon at the Γ point of monolayer WSi_2N_4 and WSi_2N_4.

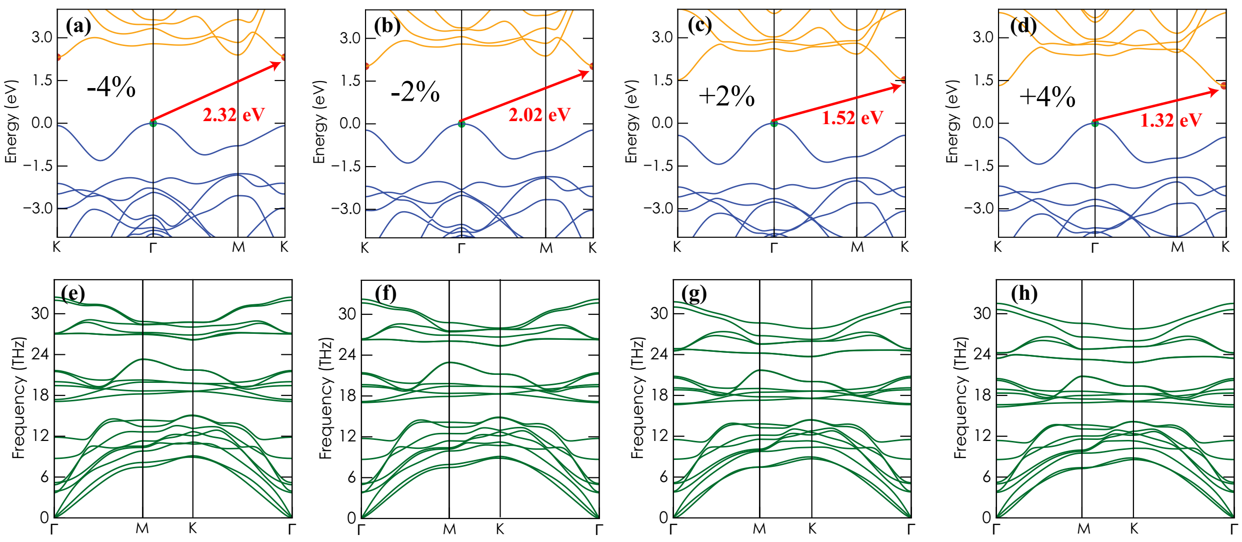

FIG. S4. Strain-dependent band structure and phonon dispersion of monolayer $MoSi_2N_4$ along *a*-axis.

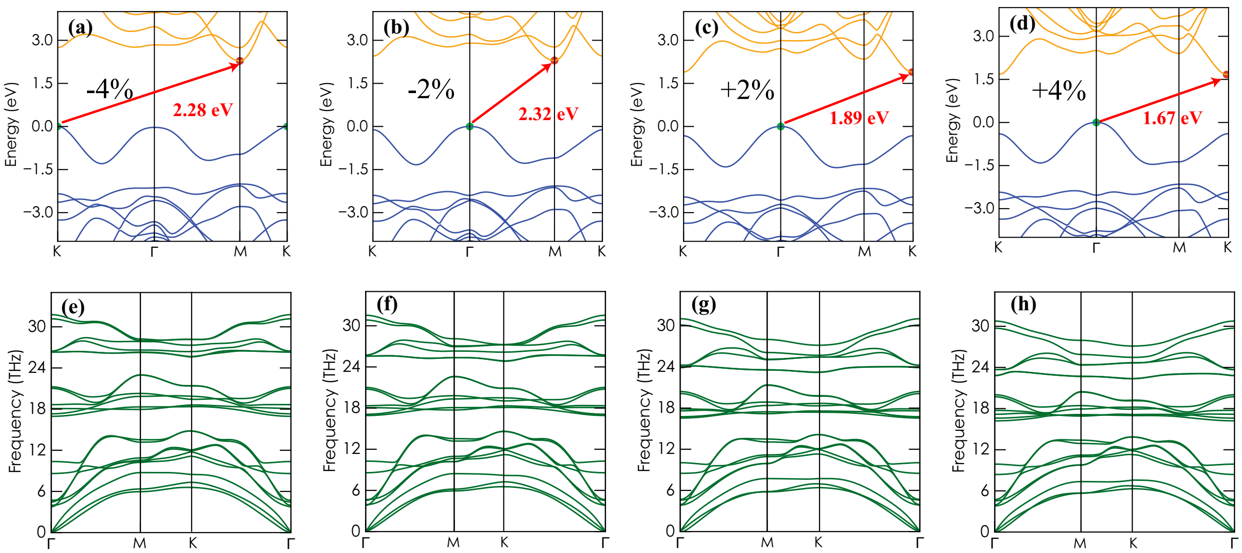

FIG. S5. Strain-dependent band structure and phonon dispersion of monolayer WSi_2N_4 along *a*-axis.

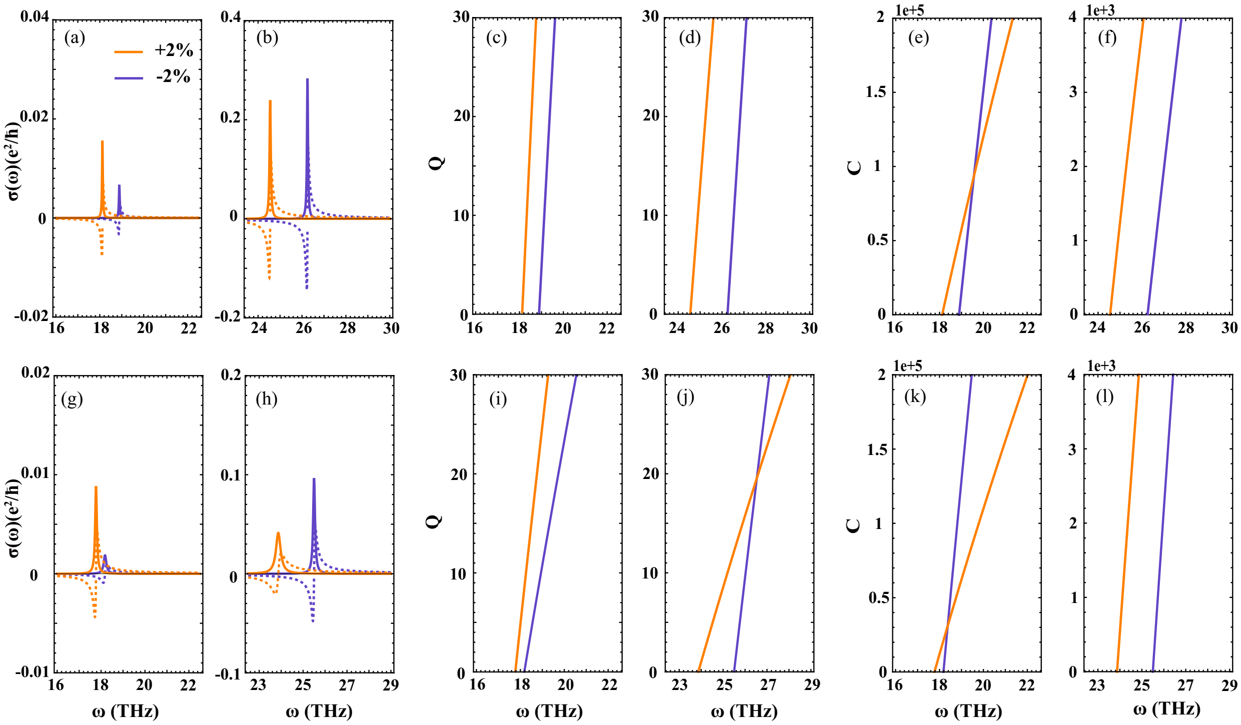

FIG. S6. Properties of strain-dependent phonon polariton of monolayer $MoSi_2N_4$ and WSi_2N_4. Real (Re σ, solid lines) and imaginary (Im σ, dashed lines) parts of the conductivity σ of 2D (a,b) $MoSi_2N_4$ and (g,h) WSi_2N_4. (c,d,i,j) Propagation-quality factor, Q = Im σ /Re σ. (e,f,k,l) Confinement factor, $C = q/q_0$.

TABLE S1. The LO phonon frequencies, the deceleration factor D and phonon damping rate γ with the consideration of strain and 1T-phase for two infrared-active LO/TO phonon modes in monolayer $MoSi_2N_4$ and WSi_2N_4.

$MoSi_2N_4$				WSi_2N_4			
strain/phase	LO [THz]	D [c]	γ [cm^{-1}]	strain/phase	LO [THz]	D [c]	γ [cm^{-1}]
-2%/2H	18.89	3.8×10^{-7}	1.6	-2%/2H	18.18	3.4×10^{-7}	5.0
-2%/2H	26.25	1.4×10^{-5}	1.9	-2%/2H	25.50	9.1×10^{-6}	3.5
0%/2H	18.21	6.3×10^{-7}	10.7	0%/2H	18.09	4.9×10^{-7}	8.3
0%/2H	25.72	1.4×10^{-5}	18.3	0%/2H	24.97	1.0×10^{-5}	16.6
+2%/2H	18.12	8.2×10^{-7}	1.4	+2%/2H	17.77	3.3×10^{-7}	3.2
+2%/2H	24.55	1.5×10^{-5}	2.3	+2%/2H	23.87	1.0×10^{-5}	8.6
0%/1T	18.20	1.9×10^{-6}	10.9	0%/1T	17.82	4.5×10^{-7}	10.9
0%/1T	25.55	1.5×10^{-5}	10.0	0%/1T	23.93	9.6×10^{-6}	10.0

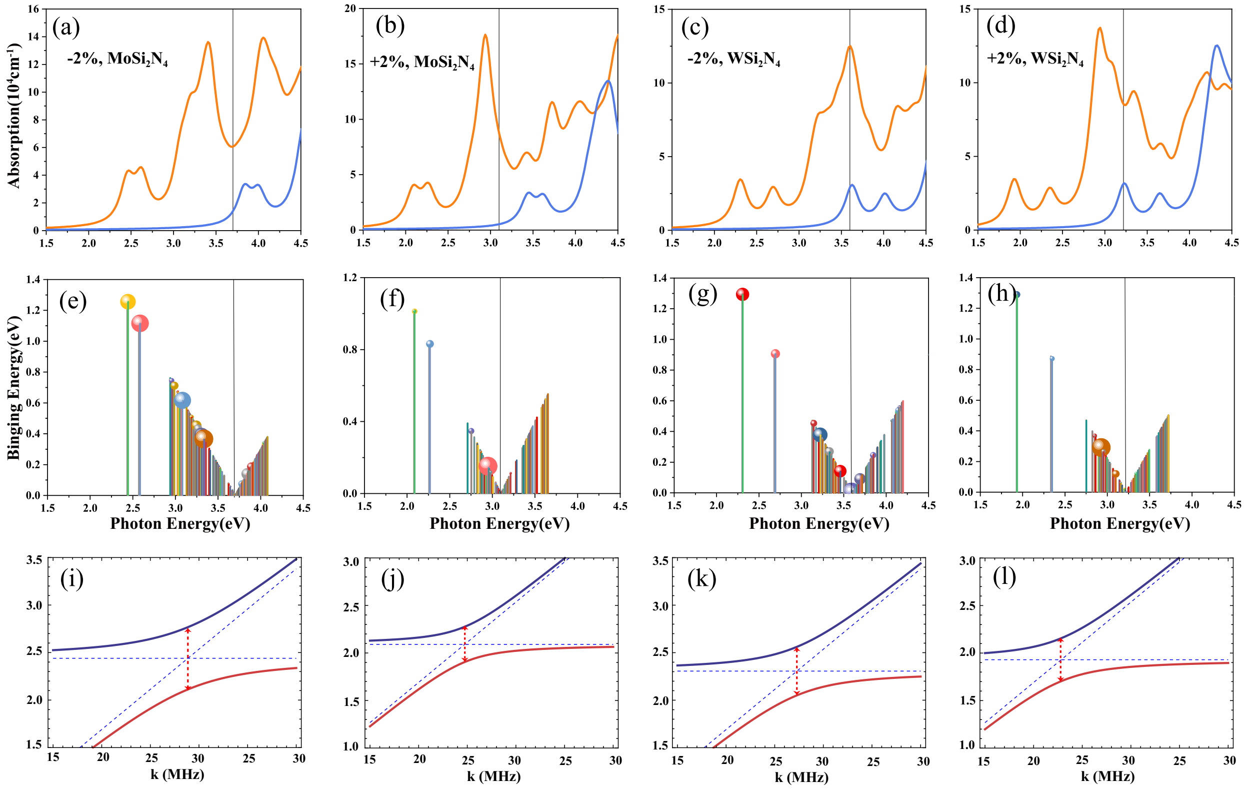

FIG. S7. Properties of strain-dependent exciton of monolayer $MoSi_2N_4$ and WSi_2N_4.

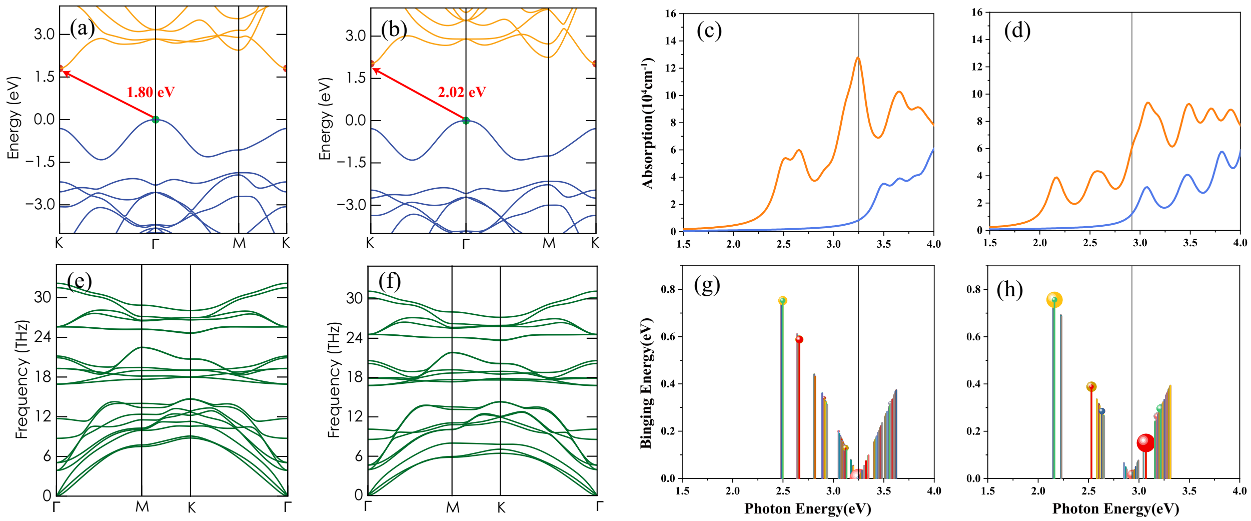

FIG. S8. Crystal and exciton properties of 1T $MoSi_2N_4$ and WSi_2N_4.
